# Therapeutic potential of naturally derived carbon dots in sepsis-associated acute kidney injury

**DOI:** 10.1186/s13020-025-01103-3

**Published:** 2025-04-11

**Authors:** Lei Wang, Zhong-Yao Li, Chong-Lei Zhong, Zi-Yang Teng, Bin Wang, Asma Rehman, Li-Wen Han, Ke-Wu Zeng, Ji-Guo Zhang, Zhi-Yuan Lu

**Affiliations:** 1 School of Pharmaceutical Sciences & Institute of Materia Medica, State Key Laboratory of Advanced Drug Delivery and Release Systems, Medical Science and Technology Innovation Center, Shandong First Medical University & Shandong Academy of Medical Sciences, Jinan, 250117 China; 2 Department of Andrology, Guang’Anmen Hospital, Chinese Academy of Chinese Medical Sciences, Beijing, 100053 China; 3 National Institute for Biotechnology & Genetic Engineering College Pakistan Institute of Engineering & Applied Sciences (NIBGE-C, PIEAS), Faisalabad, 38000 Pakistan; 4 State Key Laboratory of Natural and Biomimetic Drugs, School of Pharmaceutical Sciences, Peking University, Beijing, 100191 China

**Keywords:** Sepsis, Acute kidney injury, Carbon dots, Phytochemicals, Antioxidant, NF-κB pathway

## Abstract

**Background:**

Sepsis is a life-threatening infectious disease characterized by an uncontrolled inflammatory response and consequent multi-organ dysfunction. The kidneys, as primary excretory organs with high blood flow, are particularly susceptible to damage during sepsis. Nonetheless, the existing treatment options for sepsis-associated acute kidney injury (SA-AKI) are still restricted. Nanomedicine, especially carbon dots (CDs), has attracted considerable interest lately for outstanding biomedical characteristics.

**Methods:**

To avoid the generation of toxic effects, the natural CDs derived from *Ziziphi Spinosae Semen* (Z-CDs) were synthesized employing a hydrothermal method. The free radical scavenging capabilities of Z-CDs were evaluated by utilizing ABTS assay, NBT method, and Fenton reaction. A lipopolysaccharide (LPS)-stimulated RAW 264.7 cell model was used to explore the therapeutic potential of Z-CDs on cellular oxidative stress and inflammation. The CuSO_4_-induced zebrafish inflammation model and LPS-exposed SA-AKI mouse model were employed to assess the therapeutic efficacy of Z-CDs in vivo.

**Results:**

The synthesized Z-CDs exhibited distinctive unsaturated surface functional groups, which confer exceptional biocompatibility and the ability to scavenge free radicals. Moreover, Z-CDs were particularly effective in eliminating excess reactive oxygen species (ROS) in cells, thus protecting mitochondrial function from oxidative damage. Notably, Z-CDs have demonstrated significant therapeutic benefits in protecting kidney tissue in SA-AKI mouse model with minimizing side effects. In mechanism, Z-CDs effectively reduced ROS production, thereby alleviating inflammatory responses in macrophages through the suppression of the NF-κB pathway.

**Conclusions:**

This study developed a multifunctional nanomedicine derived from traditional medicinal herb, providing a promising pathway for the advancement of innovative drug therapies to treat SA-AKI.

**Supplementary Information:**

The online version contains supplementary material available at 10.1186/s13020-025-01103-3.

## Introduction

As a serious condition frequently encountered in clinics, sepsis is characterized by uncontrolled systemic immune responses and subsequent multi-organ dysfunction [1–3]. The excessive production of reactive oxygen species (ROS) induced by various pathogens, including bacteria and viruses, contributes to significant immunological dysregulation and oxidative damage across multiple organs [4–6]. The kidneys, which have a high blood flow, are among the most frequently impacted organs, resulting in sepsis-associated acute kidney injury (SA-AKI) that contributes to sepsis mortality [7, 8]. In particular, the excessive production of ROS in the kidneys significantly contributes to the progression of SA-AKI, resulting in oxidative damage to renal tubular epithelial cells and exacerbating inflammation [9, 10]. Following renal tubular epithelial injury, mitochondrial homeostasis undergoes dysregulation, characterized by pathological fragmentation of mitochondrial networks and ultrastructural disintegration of cristae. This organellar destabilization induces a marked surge in ROS generation [11, 12], initiating a self-amplifying cycle through NF-κB-mediated transcriptional activation that further exacerbates oxidative damage [13]. Concurrently, inflammatory mediators induce degradation of the endothelial glycocalyx in renal vasculature [14], exposing underlying adhesion molecules [15], and releasing myeloperoxidases and neutrophil extracellular traps (NETs), which directly damage the glomerular filtration barrier [16]. Notably, ferroptosis emerges as a distinct contributor to renal pathology. This iron-catalyzed process is mechanistically defined by lipoxygenase-catalyzed lipid peroxidation within polyunsaturated fatty acid-rich membranes of renal tubular cells [17]. The resultant accumulation of lipid peroxides synergistically intensifies oxidative stress, creating cross-talk between different cell death pathways and inflammatory responses that collectively drive renal parenchymal destruction [18]. In the clinic, norepinephrine [19] and fluid resuscitation [20] are the primary interventions for SA-AKI aimed at preventing kidney damage; however, they are limited by high costs, poor outcomes, and their ineffectiveness in cases of uncontrolled inflammatory responses. Consequently, there has been a substantial interest in developing novel agents that can effectively eliminate ROS and improve inflammatory responses for SA-AKI.

Carbon dots (CDs) are recognized as effective nanomedicines for treating diseases linked to ROS [21]. CDs are characterized by excellent biocompatibility, appropriate particle sizes, and controllable biological properties [22–24]. The variation in primary precursors used for the synthesis of CDs leads to the development of diverse functional groups on their surfaces, thereby enhancing the biological capabilities. Biomass-derived precursors have garnered considerable attention compared to chemical precursors due to the environmental sustainability, cost-effectiveness, and distinctive biomedical properties [25, 26]. Traditional herbal remedies, known for the diverse array of active compounds, have been employed for centuries to combat diseases and have proven to be invaluable precursors for the synthesis of novel CDs with unique properties. For example, the CDs derived from peach kernel and safflower exhibited therapeutic potential for traumatic brain injury by improving the blood–brain barrier [27]. Moreover, Lu et al. synthesized CDs using the herb *Codonopsis pilosula*, which possessed excellent erythrocyte-enhancing capabilities by regulating the JAK/STAT pathway [28]. Nevertheless, the development of cost-effective and multifunctional CDs intended to combat SA-ALI derived from various biomass materials remains a significant challenge.

**Scheme 1:**
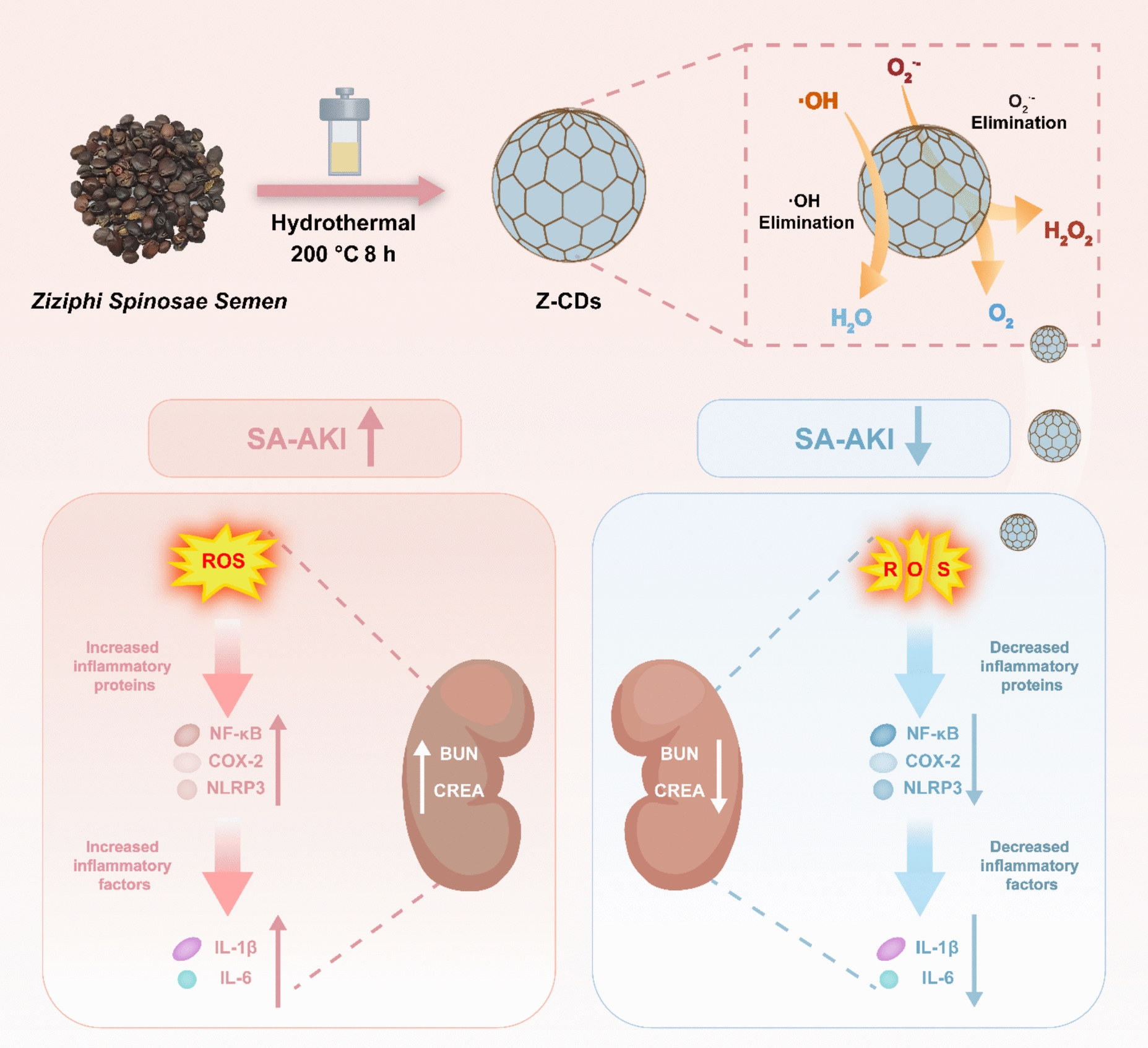
Schematic illustration demonstrates the function of Z-CDs for high ROS scavenging activity in the treatment of SA-AKI

*Ziziphi Spinosae Semen*, also known as spine date seed, is a famous traditional medicine known for its substantial dietary and therapeutic properties in nourishing the kidney [29]. The spine date seed is rich in phytochemicals, including saponins and flavonoids, which demonstrate significant bioactivities, such as anti-inflammatory effects [30]. Inspired by these insights, *Ziziphi Spinosae Semen* was chosen as a precursor for the synthesis of CDs in this study through a hydrothermal method. The synthesized CDs (Z-CDs) exhibited unique surface functional groups, providing outstanding biocompatibility and free radical scavenging capabilities (Scheme 1). Notably, Z-CDs demonstrated a notable ability to reduce excess ROS in cells, thereby protecting mitochondrial function from oxidative damage. Moreover, the Z-CDs showed notable anti-inflammatory capabilities in SA-AKI mouse model by suppressing NF-κB-dependent inflammatory pathway with minimal side effects.

In summary, our study not only developed a multifunctional and inexpensive nanomedicine derived from phytochemicals but also provided a promising direction for the development of innovative drugs based on CDs for treating SA-AKI.

## Materials and methods

### Materials

*Ziziphi Spinosae Semen* was obtained from BWT Chinese Herbal Medicine Drinks Slice (Jinan, Shandong, China) and was authenticated by Prof Li-Wen Han (Shandong First Medical University). Nitrotetrazolium Blue chloride (NBT), Riboflavin, L-Methionine, CuSO_4_, 3,3’,5,5’-tetramethylbenzidine dihydrochloride (TMB), Ibuprofen, KBr, FeSO₄·7H₂O, and NaAc-HAc (pH = 5.2) were purchased from Rhawn (Shanghai, China). The DCFH-DA probe was obtained from AmBeed (Arlington, TX, USA). The Mitosox Red was obtained from Thermo Scientific (Waltham, MA, USA). The T-AOC Assay Kit was obtained from Beyotime Biotechnology (Shanghai, China). Primary antibodies against IκBα, P65, NLRP3, COX- 2, iNOS, and GAPDH were sourced from Proteintech (Wuhan, Hubei, China), whereas antibodies specific for p-IκBα and p-P65 were purchased from Cell Signaling Technology (Boston, MA, USA). The LPS and DMSO were sourced from Sigma-Aldrich (Shanghai, China). More detailed information about the chemicals and reagents used in this study is provided in Supplementary Table S1.

### Preparation of Z-CDs

*Ziziphi Spinosae Semen* fragments were soaked in deionized water and heated in an autoclave at 200 °C for 8 h. The mixture was subjected to filtration with a 0.22 μm membrane after cooling to room temperature. Subsequently, this solution underwent dialysis for 10 h using a 500 Da dialysis membrane and was subsequently subjected to freeze-drying to obtain Z-CDs. The detailed synthesis process is shown in Figure S1.

### Characterization of Z-CDs

A Talos F200S Transmission Electron Microscope (Thermo Scientific, Waltham, MA, USA) was employed to characterize the morphology and structure of the Z-CDs. The measurement of the Z-CDs thickness was conducted with a Dimension Icon AFM (Bruker, Karlsruhe, Germany). The FTIR data were captured with a Thermo Scientific FT-IR spectrophotometer (Waltham, MA, USA) over a wavelength range of 400–4000 cm^−1^. X-ray Photoelectron data was collected using a Thermo Scientific K-Alpha X-ray Spectrometer (Thermo Scientific, Waltham, MA, USA). A Hitachi F- 7000 spectrometer (Hitachi, Tokyo, Japan) was used to record the fluorescence spectra. Data from X-ray diffraction (XRD) were gathered with a Rigaku D/MAX- 2600 (Rigaku, Tokyo, Japan) at a scanning speed of 2° per min.

### The total antioxidant capacity assay

The T-AOC Assay Kit was used to assess the overall antioxidant capacity of Z-CDs, according to the manufacturer's guidelines. ABTS^•+^ radical cation exhibits a characteristic absorption peak at 414 nm. The absorbance changes of Z-CDs at different concentrations were monitored over 90 s at 414 nm. This allowed for the determination of the remaining ABTS^•+^ concentration. Continuous spectral scanning was performed to obtain the visible light absorption spectra for each concentration group.

### Superoxide anion scavenging assay

The scavenging effect of Z-CDs on O_2_^•−^ was detected by the NBT method. In brief, the solution containing Z-CDs (0–400 μg/mL), NBT (0.05 mM), L-met (13 mM), and riboflavin (20 μM) in a 25 mM PBS buffer (pH 7.4) was exposed to LED light for 3 min. The removal effect of O_2_^•−^ is gauged by the variation in absorbance at 560 nm.

### Hydroxyl radical scavenging activity assay

The evaluation of Z-CDs property against •OH radicals was conducted as reported with slight modifications [31]. The Z-CDs (100 μL) at varying concentrations were mixed with 20 μL FeSO_4_·7H_2_O (10 mM), 65 μL H_2_O_2_ (100 mM), 10 μL NaAc-Hac buffer (pH 5.2) and 5 μL TMB (10 mM) solution. After reacting for 60 min in the dark, the absorbance at 652 nm was checked, and the eliminating rate was evaluated using an equation:

Eliminating rate% = (A_TMB_-A _Sample_)/A_TMB_ × 100%

where A refers to the absorbance at 652 nm in solutions that include and exclude Z-CDs samples.

### Measurement of intracellular ROS

Using a DCFH-DA probe, the measurement of ROS in RAW264.7 cells was conducted, according to the instructions from AmBeed (Arlington, TX, USA). In short, cells were incubated with the probe in a serum-free medium for 30 min. After washing three times with PBS, an LSM980 confocal microscope (Zeiss, Oberkochen, Baden-Württemberg, Germany) was used to capture the fluorescence images, with optimal excitation at 492 nm and emission at 517 nm.

For the assessment of superoxide production by mitochondria, the MitoSOX Red Indicators were employed according to instructions. The probe, diluted to 500 nM, was incubated with cells for 30 min at 37 °C, without light. After washing three times with PBS, the fluorescence images were obtained using an LSM980 confocal microscope, with optimal absorption at 396 nm and emission at 610 nm.

### CuSO₄-induced zebrafish model

The transgenic zebrafish strain Tg (lyz: DsRED2) was utilized and kept as outlined in earlier descriptions [32]. In short, 20 zebrafish embryos (72 h of post-fertilization) were placed in a six-well plate and randomly distributed into the indicated groups, including the control, model, positive control (4 μg/mL ibuprofen), and Z-CDs at 50, 100, and 200 μg/mL. After 6 h of pre-protection for the positive control and Z-CDs groups, CuSO_4_ (20 μM) was employed to provoke inflammation for 2 h. Afterward, ten zebrafish were chosen at random to examine neutrophil distribution with a fluorescent microscope. All experiments were sanctioned by the Laboratory Animal Ethical and Welfare Committee at the Institute of Materia Medica, Shandong Academy of Medical Sciences (No. 202405).

### In vivo evaluation in an SA-AKI mouse model

BALB/C male mice, 18–20 g and 7–8 weeks old, were sourced from Charles River (Beijing, China), and maintained free access to water and food under standard SPF conditions at 25 °C and 50% humidity. For the therapeutic validation of Z-CDs in an SA-AKI mouse model, random division of the mice resulted in five groups: control, LPS, LPS + DEX (3 mg/kg), LPS + Z-CDs (25 mg/kg), and LPS + Z-CDs (50 mg/kg). To establish the SA-AKI mouse model, mice were given an intraperitoneal injection of LPS at a concentration of 5 mg/kg. Then, the mice were intraperitoneally administrated with 100 μL PBS with or without Z-CDs (25 or 50 mg/kg) after LPS injection for 1 h. All animals were euthanized with isoflurane anesthesia after 24 h, and the tissue samples were obtained for further analysis.

For biosafety assessment of Z-CDs, the mice were intraperitoneally injected with saline or Z-CDs (100 and 500 mg/kg). The weight of each mouse was monitored daily for a week. Blood analysis was performed using a hematology analyzer from the mice after Z-CDs injection. The serum biochemical indicators, including creatinine (CREA), aspartate aminotransferase (AST), blood urea nitrogen (BUN), and alanine aminotransferase (ALT) were analyzed using a liver and kidney function activity assay kit. The organs mentioned were subjected to H&E staining following the guidelines. Approval for all animal studies was granted by the Ethics Committee of the Experimental Animal Centre of Shandong First Medical University (No. W202410180685).

For more experimental details, please refer to Supporting Information.

### Statistical analysis

Statistical analysis was performed using Prism 10 (GraphPad Software). Data are presented as mean ± SD unless stated otherwise. *P* values were determined using an unpaired two-tailed Student's t-test, and values below 0.05 were considered statistically significant.

## Results

### Synthesis and characterization of Z-CDs

**Fig. 1:**
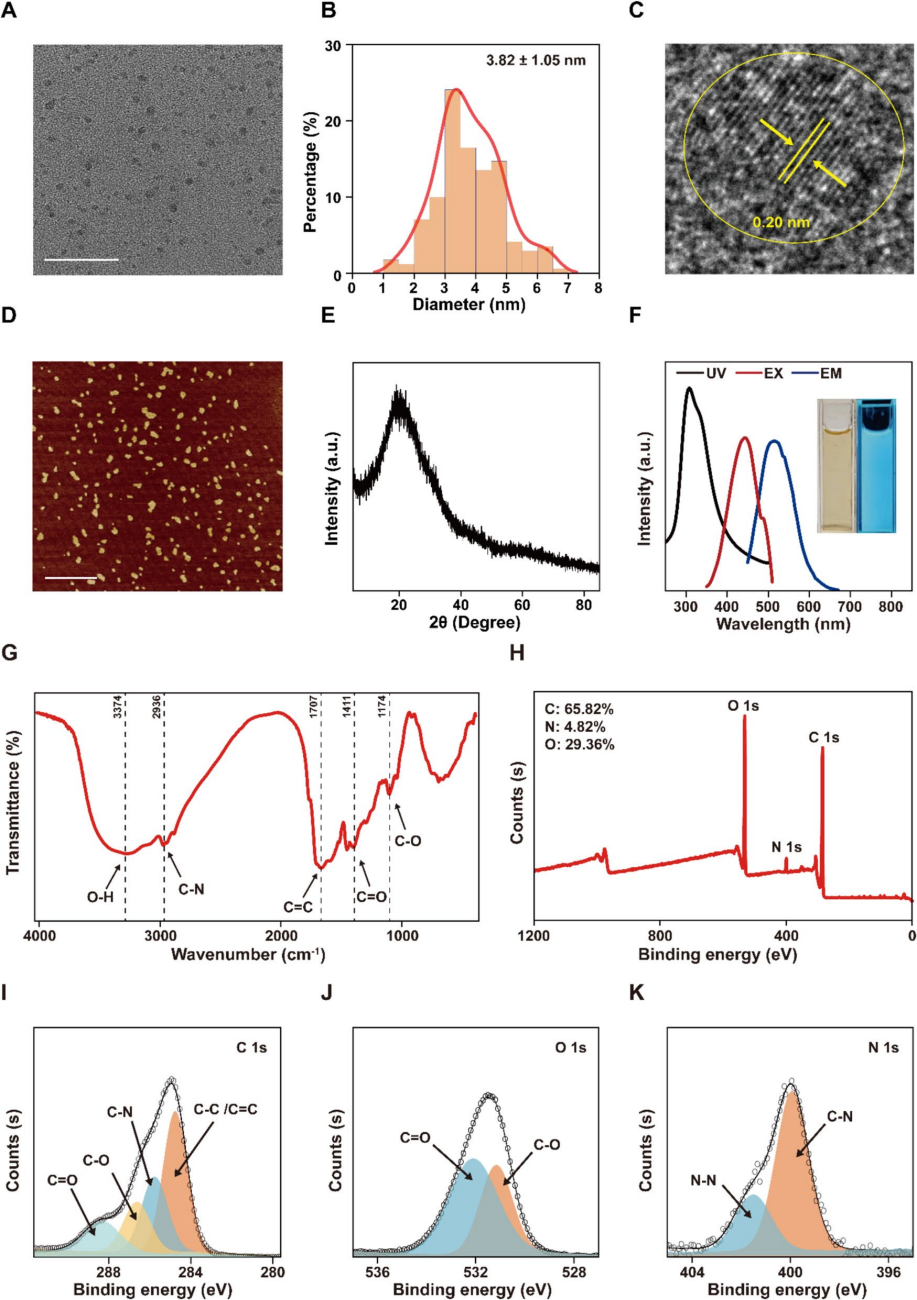
Characterization of Z-CDs. **A** TEM image of Z-CDs. Scale bar: 50 nm. **B** Size distribution of Z-CDs. **C** The high-resolution TEM image of Z-CDs. **D** AFM image of Z-CDs. Scale bar: 600 nm. **E** XRD pattern of Z-CDs. **F** Ultraviolet absorption spectra (black line), fluorescence excitation (red line), and emission (blue line) spectra of Z-CDs. Inset: Images of Z-CDs taken under daylight (left) and 365 nm UV lamp (right). **G** FTIR spectra of Z-CDs. **H** Full scan XPS spectrum of Z-CDs. **I–K** High-resolution C 1 s, O 1 s, and N 1 s XPS spectra of Z-CDs

To avoid the generation of toxic effects, we employed a hydrothermal method to synthesize Z-carbon dots (Z-CDs) based on spine date seed at 200 °C for 8 h (Figure S1, Supporting Information). TEM images demonstrated that the Z-CDs possessed high uniformity and good dispersibility, featuring an average size of 3.82 ± 1.08 nm (Fig. 1A and B). The high-resolution TEM image further corroborated the successful synthesis of the Z-CDs, revealing a lattice fringe of 0.2 nm (Fig. 1C), which corresponded to the crystalline plane of carbon [33]. By employing atomic force microscopy (AFM), we further analyzed the morphology of Z-CDs. As shown in Fig. 1D, Z-CDs displayed an even distribution and spherical shape with uniform thickness. The XRD analysis confirmed the purity of the synthesized Z-CDs, exhibiting a diffraction peak at 22°, indicative of amorphous carbon structures (Fig. 1E). In the ultraviolet absorption spectra of Z-CDs (Fig. 1F), a stronger peak was observed at approximately 300 nm, resulting from the π-π* transition in the sp^2^ structural domain [34]. By using an integrating sphere system, the absolute quantum yield of the Z-CDs was determined as ≈5.3%, indicating the bioimaging potential of Z-CDs. Simultaneously, Z-CDs demonstrated excitation-dependent characteristics, with an excitation wavelength peak at 440 nm and an emission wavelength of 530 nm (Fig. 1F). The optical characteristics of Z-CDs were additionally validated through three-dimensional fluorescence emission spectra (Figure S2, Supporting Information), which remained stable over various NaCl concentrations (0–1.0 M) (Figure S3, Supporting Information). As depicted in the laser confocal scanning microscopy (LSM980 confocal microscope) images, Z-CDs labeled RAW264.7 cells efficiently within 5 min under 405 nm excitation (Figure S4, Supporting Information), resulting in blue fluorescence that remained stable for at least 1 h. These results underscored the potential of utilizing Z-CDs for cellular imaging in biological applications.

To explore the surface structure and chemical composition of the Z-CDs, we employed FTIR and XPS analysis. The FTIR spectrum result in Fig. 1G detected stretching bands for O–H at 3374 cm^−1^, C–N at 2936 cm^−1^, C = C at 1707 cm^−1^, and C = O at 1611 cm^−1^. The presence of hydrophilic groups such as O–H contributed to the excellent water solubility of Z-CDs. Moreover, XPS analysis revealed that Z-CDs primarily consisted of C (65.82%), N (4.82%), and O (29.36%) (Fig. 1H). The C 1 s spectrum with high resolution was divided into four major peaks, which represented C = O at 288.34 eV, C–N at 285.74 eV, C–O at 286.64 eV, and C = C/C–C at 284.74 eV. (Fig. 1I). Two principal peaks at 531.2 and 532.1 eV were identified in the O 1 s band deconvolution, representing C-O and C = O (Fig. 1J). The N 1 s band deconvolution revealed two primary peaks at 399.8 and 401.6 eV, indicating the presence of C-N and N–N (Fig. 1K). Therefore, the synthesized Z-CDs demonstrated distinctive biological functions attributed to the appropriate particle size, along with the structural and optical properties.

### Z-CDs exhibit distinctive free radical scavenging capabilities in vitro

**Fig. 2:**
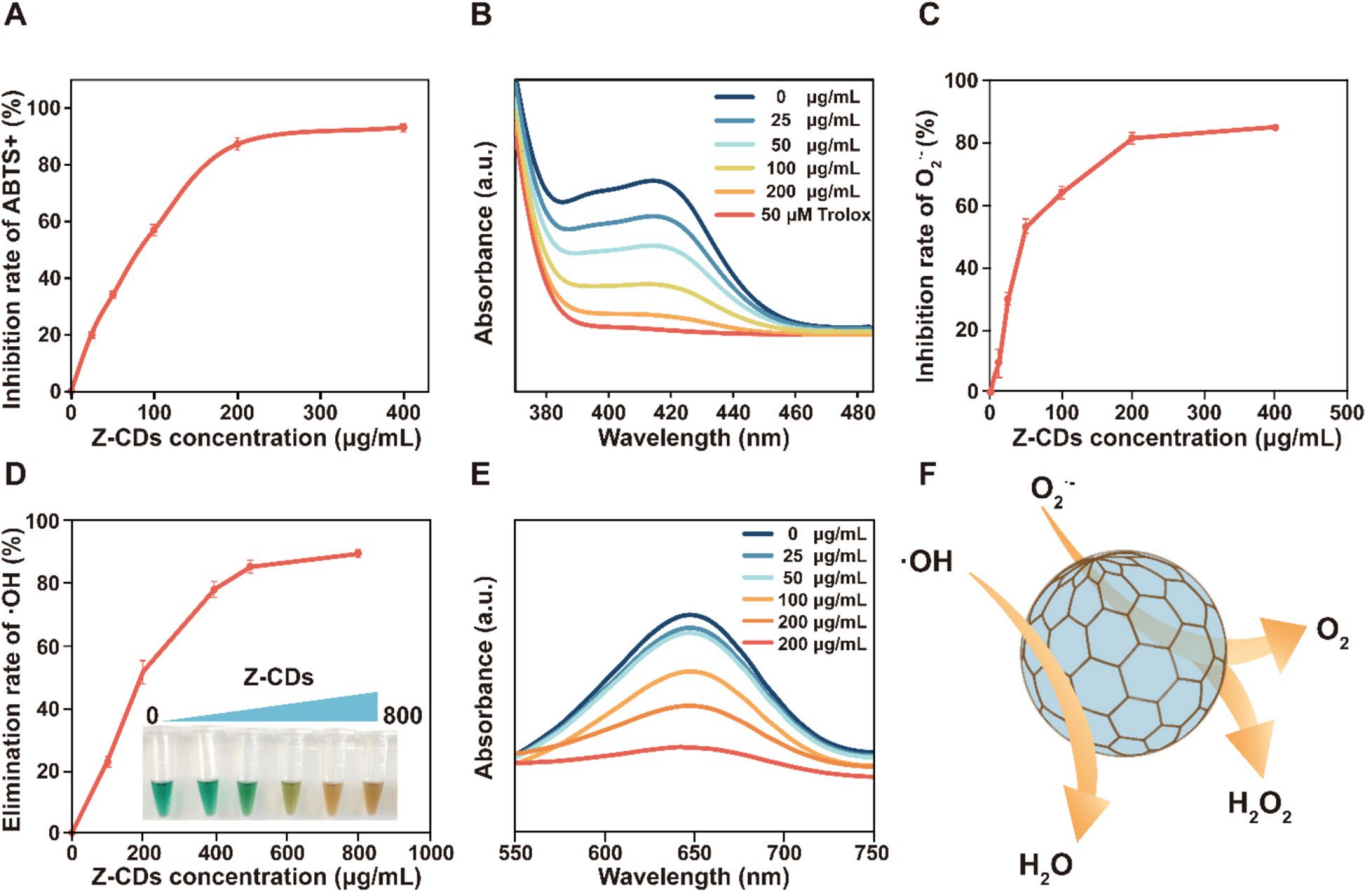
Z-CDs exert excellent ROS scavenging activities of in vitro. **A** The total antioxidant capacity of Z-CDs was measured by using the ABTS method. **B** The UV–vis absorption spectra of ABTS^•+^ solutions after mixing with Z-CDs at different concentrations. **C** The elimination O2^•−^ capacity of Z-CDs of different concentrations. **D** The •OH scavenging capacity of Z-CDs was detected based on the Fenton reaction. **E** UV–vis absorption spectra of TMB solutions after mixing with Z-CDs. **F** Schematic diagram of the free radicals scavenging activity of the Z-CDs. Data are expressed as mean ± SD, n = 3

Previous research has indicated that unsaturated groups impart significant antioxidant properties to biomass-derived CDs [31]. Thus, the presence of unsaturated groups in Z-CDs motivated us to test whether the Z-CDs possessed antioxidant capabilities. First, the total antioxidant capacity of Z-CDs was evaluated by using the ABTS assay. As shown in Fig. 2A and B, the production of oxidized ABTS was significantly inhibited by Z-CDs, with an inhibition rate exceeding 80% at a concentration of 200 μg/mL. This inhibition resulted in an antioxidant effect comparable to that of 50 μM Trolox, thereby demonstrating the remarkable antioxidant capacity of Z-CDs. Given that the cellular ROS, which are by-products of aerobic metabolism, primarily consists of hydrogen peroxide (H_2_O_2_), hydroxyl radicals (•OH), and superoxide anion (O_2_^•−^) [35], we evaluated the scavenging ability of Z-CDs for these free radicals, respectively. We initially evaluated the O_2_^•−^ scavenging ability of Z-CDs using nitrotetrazolium blue chloride (NBT) as an indicator. NBT can be reduced by O_2_^•−^ to generate a product that exhibits a distinct absorbance peak at 560 nm [36]. Here, Z-CDs displayed a concentration- dependent effectiveness in eliminating O_2_^•−^ (Fig. 2C). To investigate the efficiency of hydroxyl radical removal by Z-CDs, we employed a Fenton method that combined Fe^2+^ with H_2_O_2_ to generate •OH. Quantitative analysis demonstrated that Z-CDs effectively eliminated the •OH (Fig. 2D and E), indicating the enhanced capacity as antioxidants. In addition, Z-CDs failed to neutralize H_2_O_2_ directly (data not shown), showing an absence of CAT-like activity. Therefore, our study revealed that the capacity of Z-CDs to scavenge O_2_^•−^ and •OH contributed to the elimination of ROS (Fig. 2F).

### Z-CDs alleviate cellular oxidative damage by eliminating mitochondrial ROS

**Fig. 3:**
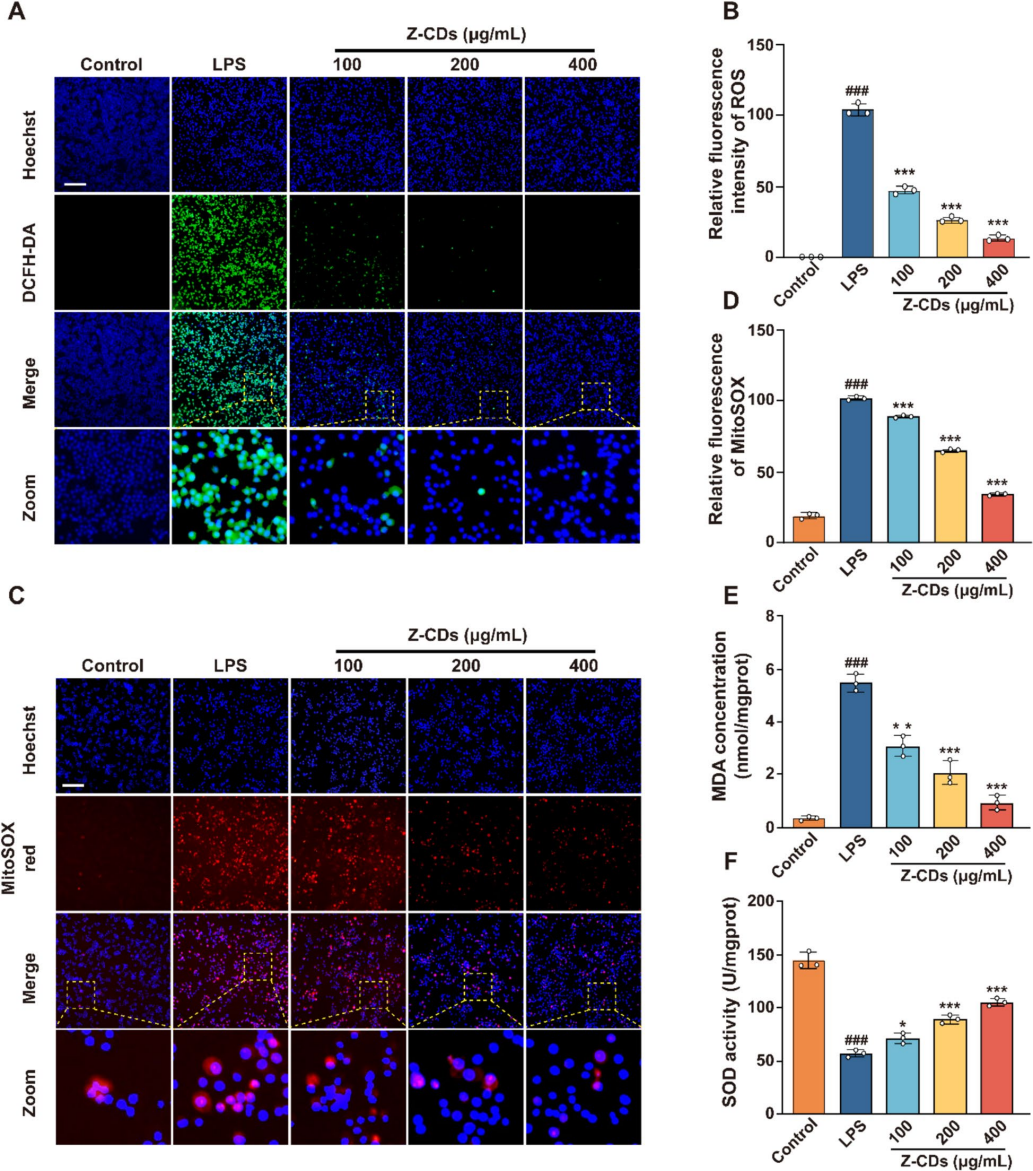
Z-CDs alleviate cellular oxidative damage by eliminating mitochondrial ROS. **A, B** Detection of cellular ROS in RAW 264.7 cells with or without Z-CDs for 24 h using DCFH-DA probe. Scale bar: 20 μm. **C, D** The mitochondrial superoxide in RAW 264.7 cells were measured by using MitoSOX red probe. Scale bar: 20 μm. **E, F** Assessment of cellular MDA and SOD levels. Data are expressed as mean ± SD, n = 3. ^*###*^*P* < 0.001, vs. control group; *P < 0.05, **P < 0.01, ***P < 0.001, vs LPS group

The remarkable ability of Z-CDs to scavenge ROS in vitro prompted us to investigate its effect in depleting cellular ROS. Since infection-triggered excessive ROS and unregulated inflammatory responses are key to SA-AKI progression, we utilized a lipopolysaccharide (LPS)-stimulated RAW 264.7 cell model to explore the therapeutic potential of Z-CDs on cellular oxidative stress and inflammation [37]. As shown in Fig. 3A and B) LPS treatment led to a significant rise in cellular ROS, which was reduced by Z-CDs in a concentration-dependent manner, showing the high efficiency in scavenging cellular ROS. Since mitochondria are the primary site for ROS production [38], we then monitored mitochondrial superoxide (mtROS) levels. As expected, the LSM980 confocal microscope images showed that Z-CDs notably inhibited the LPS-induced ROS production in mitochondria (Fig. 3C, D). Given that excessive ROS can cause irreversible oxidative damage, we subsequently evaluated the protective effects of Z-CDs against oxidative damage by measuring the levels of cellular superoxide dismutase (SOD) and malondialdehyde (MDA). We observed that Z-CDs significantly boosted SOD activity while lowering MDA levels (Fig. 3E, F), highlighting the protective role of Z-CDs in oxidative damage. Thus, Z-CDs showed a remarkable ability to clear excess ROS in cells, thus preserving mitochondrial function from oxidative damage.

### Z-CDs exert anti-inflammatory effects by inhibiting the NF-κB pathway

**Fig. 4:**
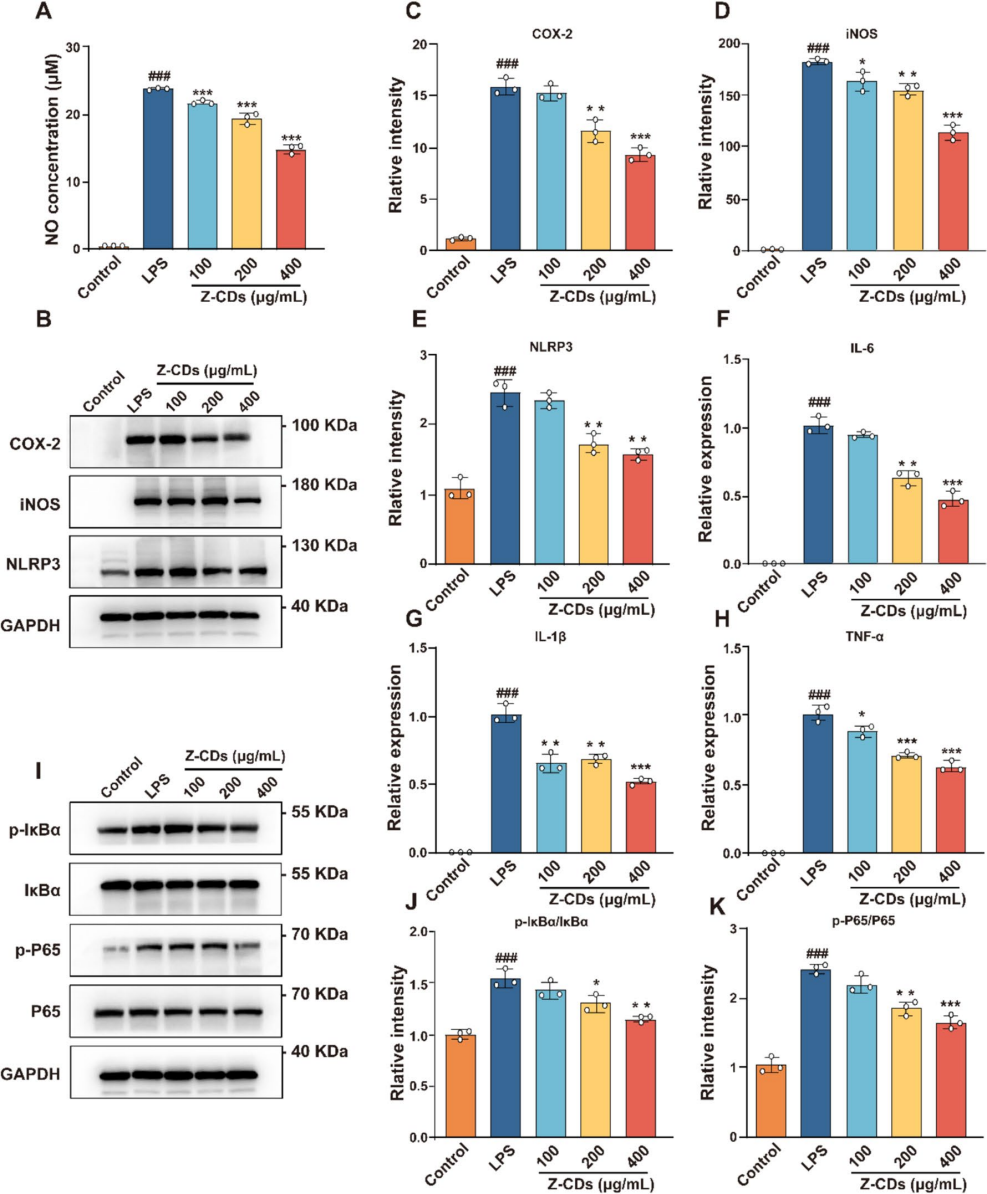
Z-CDs exert anti-inflammatory effects by inhibiting the NF-κB pathway. **A** Quantification of the NO production in RAW 264.7 cells after being administered with Z-CDs. **B** Representative images of COX- 2, iNOS, and NLRP3 protein levels in RAW 264.7 cells. **C–E** Quantitative analysis of the indicated protein levels in (**B**). **F–H** The expression of IL- 6, IL- 1β, and TNF-α were measured by qRT-PCR. **I–K** Representative profiles and quantitative analysis of phosphorylated IκBα (Ser32) and phosphorylated P65 (Ser536) with or without Z-CDs treatment. Data are expressed as mean ± SD, n = 3. ^*###*^*P* < 0.001, vs. control group; *P < 0.05, **P < 0.01, ***P < 0.001, vs. LPS group

Given the association between excessive ROS and inflammatory diseases [39, 40], we proceeded to explore the anti-inflammatory effects of Z-CDs due to the remarkable antioxidant capacity. As shown in Fig. 4A, the LPS-induced production of nitric oxide was notably decreased by Z-CDs after 24 h treatment, with no toxic effects on cells. Meanwhile, the levels of pro-inflammatory proteins, including COX- 2, iNOS, and NLRP3, were reduced in a concentration-dependent manner by Z-CDs (Fig. 4B–E). Since LPS exposure induces macrophages undergoing M1 polarization and producing pro-inflammatory cytokines to combat pathogen [41], we then examined the transcription of pro-inflammatory cytokines using qRT-PCR. The treatment with Z-CDs for 6 h led to a significant reduction in the expression of TNF-α, IL- 6, and IL- 1β in a manner that was comparable to the previous study (Fig. 4F–H). The activation of the TLR4/NF-κB pathway regulates the production of inflammatory cytokines in RAW 264.7 cells in response to LPS [42]. Consequently, we measured the levels of key proteins in RAW 264.7 cells, both with and without treatment with Z-CDs. Figure 4I–K showed that LPS significantly increased the levels of phosphorylated IκBα and p65, which were significantly reversed by Z-CDs. Therefore, our findings indicated the therapeutic potential of Z-CDs in inflammatory diseases by modulating the TLR4/NF-κB pathway.

### Z-CDs exert a noteworthy therapeutic benefit against SA-AKI in vivo by eliminating ROS and improving inflammatory response

**Fig. 5:**
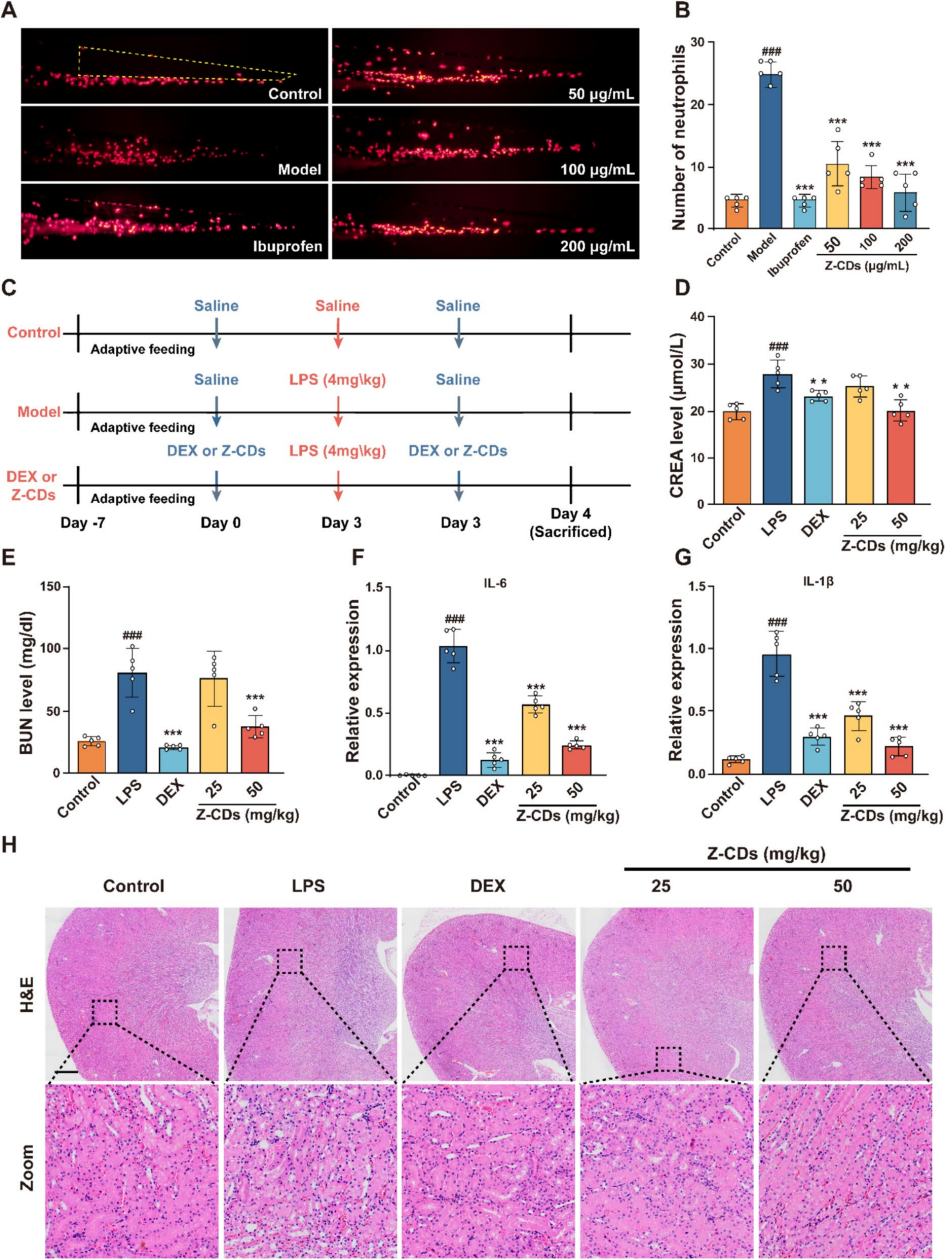
Z-CDs exert as a therapeutic agent against SA-AKI in vivo by eliminating ROS and improving inflammatory response. **A** Phenotypes and quantitative analysis of neutrophil distribution and tail neutrophil spread to lateral line in zebrafish. The yellow box indicates the neutrophil spreading area (neutrophils of 72 hpf transgenic zebrafish exhibiting red fluorescence). **B** Quantification of the number of neutrophils from (**A**). Each dot represents one zebrafish, n = 5. **C** Schematic diagram of the animal experimental program. **D–E** Quantitative determination of serum levels of CREA and BUN, indicators of kidney impairment in mice. Each dot represents one mouse, n = 5. **F–G** Expression of mRNA levels of IL- 6 and IL- 1β, inflammation-related indicators, in mouse kidney tissues by qPCR. n = 5. **H** Representative images of the histologic morphology of kidney tissue assessed by H&E staining. n = 5. Scale bar: 100 μm. Data are expressed as mean ± SD. ^###^*P* < 0.001, vs. control group; *P < 0.05, **P < 0.01, ***P < 0.001, vs. LPS group

The ROS scavenging and anti-inflammatory capacities encouraged us to evaluate the therapeutic potential of Z-CDs against inflammatory disease in vivo*.* Initially, we assessed the therapeutic efficacy of Z-CDs using a widely established zebrafish model of inflammation induced by CuSO_4_ [32]. Zebrafish embryos, 48 h post-fertilization, were subjected to a 6-h pretreatment with Z-CDs, 0.1% DMSO (used as a solvent control), and 20 µM ibuprofen (serving as a positive control). Then, the embryos were exposed to 20 µM CuSO_4_ for 2 h to induce oxidative damage and promote the infiltration of neutrophils (Figure S5, Supporting Information). The administration of Z-CDs resulted in a significant reduction in neutrophil migration in a concentration-dependent manner (Fig. 5A, B), indicating the potential as a promising candidate for treating inflammatory-related diseases. Next, we proceeded to assess the therapeutic effects of Z-CDs in a mouse model (Fig. 5C). Following the intraperitoneal injection of LPS, the mice displayed symptoms such as tremors, squinting, and increased secretions within 24 h, which were absent in the mice treated with dexamethasone or Z-CDs. To investigate the kidney-protective effects of Z-CDs, serum CREA and BUN levels were monitored. As shown in Fig. 5D and E, the administration of Z-CDs at a dose of 50 mg/kg effectively reduced the LPS-induced elevation of serum CREA and BUN, achieving a therapeutic effect similar to that of dexamethasone. Meanwhile, the expression of cytokines in the kidney tissues was significantly reduced following treatment with Z-CDs (Fig. 5F and G). Importantly, the H&E staining images of kidney tissues further revealed that Z-CDs prevented the inflammatory infiltration and severe damage of renal tubular epithelial cells (Fig. 5H). These findings indicate that Z-CDs have a therapeutic effect on SA-AKI, demonstrating considerable potential for translational medicine.

### Z-CDs ehibit excellent biocompatibility and safety

**Fig. 6:**
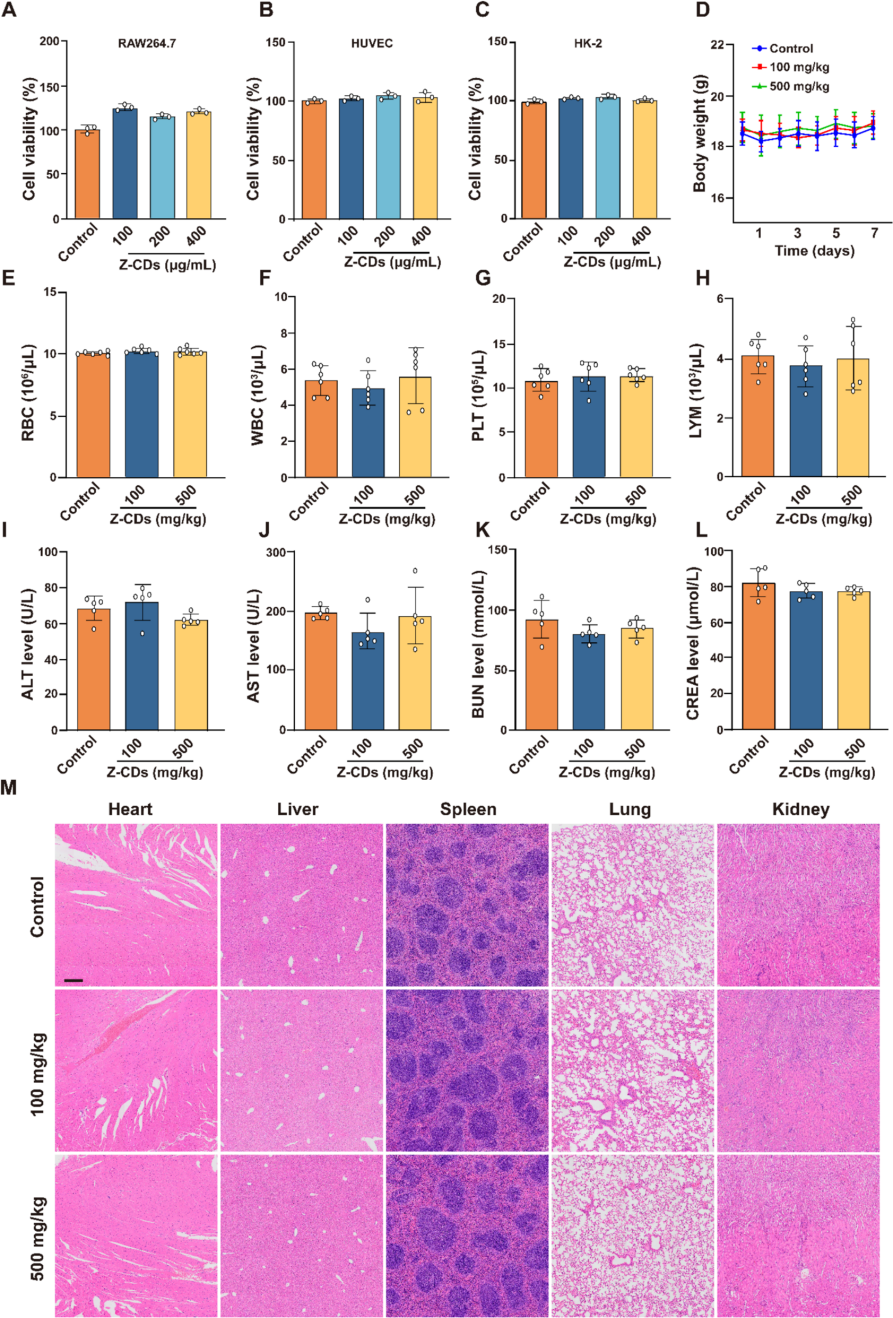
Biosafety valuation of Z-CDs. **A–C** CCK- 8 analysis was performed with various concentrations of Z-CDs in RAW264.7, HUVEC, and HK2 cells. n = 3. **D** Quantitative analysis of body weight. n = 6. **E–H** Analysis of peripheral blood in indicated mice. n = 6. **I–L** Serum levels of ALT, AST, CERA, BUN in indicated mice. n = 5. **M** Representative images of the histologic morphology of different tissues (heart, liver, spleen, lungs, and kidneys) were evaluated by H&E staining. n = 5. Scale bar: 100 μm. Data are expressed as mean ± SD

To address the clinical translational limitations related to the biosafety of Z-CDs, we conducted an evaluation of the biological toxicity. First, we assessed the cytotoxicity of the Z-CDs using RAW264.7, HUVEC, and HK- 2 cell lines, which represent human immune and epithelial cells. The results from the CCK- 8 assay revealed an absence of cytotoxic effects toward any of these cell lines at a concertation of 400 µg/mL (Fig. 6A–C). Subsequently, we conducted an in vivo biosafety assessment by administering Z-CDs at doses of 100 mg/kg and 500 mg/kg body weight to mice. By monitoring body weight changes every day, no considerable body weight differences were noted after a week of Z-CDs administration (Fig. 6D). Analysis of peripheral blood in mice revealed no significant abnormalities (Fig. 6E–H). Meanwhile, the serum biochemical indicators, such as AST, ALT, CREA, and BUN, did not show any noticeable differences between these groups (Fig. 6I–L), highlighting the absence of hepatorenal toxicity associated with Z-CDs. Furthermore, H&E staining was also employed to evaluate the toxicity in the heart, liver, spleen, lung, and kidney. The histopathological analysis revealed no significant abnormalities across any of the groups (Fig. 6M). Thus, these findings suggested that Z-CDs showed acceptable biosafety.

## Discussion

The development of effective and biocompatible therapeutic agents for sepsis-associated acute kidney injury (SA-AKI) remains a critical challenge due to the complex interplay of oxidative stress and uncontrolled inflammation driving the pathology. In this study, we successfully synthesized multifunctional carbon dots (Z-CDs) derived from *Ziziphi Spinosae Semen* and demonstrated their remarkable therapeutic potential in mitigating SA-AKI through dual mechanisms of ROS scavenging and anti-inflammatory action. Our findings highlight the promise of biomass-derived nanomaterials as innovative, cost-effective, and sustainable solutions for managing inflammatory and oxidative stress-related diseases.

As a traditional medicine known for nourishing the kidney and mind, hundreds of compounds have been identified from *Ziziphi Spinosae Semen*, including flavonoids, saponins, and polysaccharides, with various pharmacological effects [30]. Interestingly, spectroscopic characterization (FTIR/XPS) revealed surface functionalization of Z-CDs with hydroxyl, carbonyl, and amino moieties, suggesting successful integration of the herb's native phytoconstituents (flavonoids, saponins) into the carbon nanostructure during thermal degradation [43]. Crucially, the high-pressure hydrothermal environment facilitated the structural reorganization of polar functional groups (-OH, -COOH) into heteroatom-doped (e.g., N, O) carbon matrices while preserving electron-rich oxygen clusters essential for redox modulation. These architecturally conserved active sites enable potent ROS neutralization (O₂•⁻, •OH) through π-electron cloud interactions, demonstrating significantly enhanced scavenging efficiency compared to honeysuckle-derived counterparts [34, 44]. Notably, Z-CDs demonstrated superior antioxidant capacity compared to synthetic antioxidants like Trolox in vitro, underscoring their potential as therapeutic agents for oxidative stress-related conditions [45]. Additionally, the phytochemical diversity of the herbs would endow the generated carbon dots distinctive bioactivities. For example, the *Codonopsis pilosula* derived carbon dots, which is enriched with polysaccharides, exhibited excellent erythrocyte-enhancing capabilities as a superoxide dismutase (SOD) like nanozyme [28]. Yang et al. developed novel functional carbon dots using carbonized Platycladus orientalis as precursor, which accelerate hemostasis through activation of platelets [46]. Thus, the methodology exemplifies a rational fusion of pharmacognostic wisdom with nanoscale engineering, establishing a blueprint for next-generation phytomedicine-derived nanotherapeutics.

A key finding of this study is the ability of Z-CDs to protect mitochondrial function by reducing mitochondrial ROS (mtROS) in LPS-stimulated macrophages. Mitochondria are both primary sources and targets of ROS in inflammatory conditions, and their dysfunction exacerbates cellular damage and inflammation [29]. Our results revealed that Z-CDs effectively ameliorated oxidative stress by preserving mitochondrial ultrastructure, enhancing superoxide dismutase activity, and reducing lipid peroxidation markers as quantified in Fig. 3, indicating their critical role in mitigating SA-AKI progression. Consistently, Z-CDs administration significantly alleviated H_2_O_2_-induced oxidative stress in renal proximal tubular epithelial cells by scavenging intracellular ROS, followed by reduced MDA levels and increased CAT activity (Figure S6, Supporting Information). Importantly, Z-CDs exerted potent inhibitory effects on NF-κB nuclear translocation through dose-dependent suppression of IκBα degradation and p65 phosphorylation, suggesting selective disruption of the TLR4/NF-κB signaling axis—a central mediator of sepsis-induced organ dysregulation [33]. While our phosphoproteomic analysis confirmed NF-κB pathway modulation, a critical knowledge gap remains regarding upstream regulatory targets of Z-CDs. Li et al. reported a ginger-derived carbon dots accelerate wound healing by effectively blocking the TLR4-mediated NF-κB pathway, providing direct phenotypic evidence for the abilities of carbon dots on regulating TLR4 activity [47]; Given that the nano-protein interaction helps to modulate the structure of target proteins [48], we cannot dismiss the possibility that Z-CDs may target TLR4 or MyD88 directly and regulate their protein activity, which deserves future investigations. Importantly, the anti-inflammatory effects of Z-CDs in vivo mirrored those of dexamethasone, a potent glucocorticoid, but without the associated side effects, as evidenced by biosafety assessments. This mechanism is particularly relevant in SA-AKI, where excessive inflammation and oxidative stress synergistically damage renal tubules.

Nonetheless, there are still several questions remain to be addressed. First, while the surface functional groups of Z-CDs are implicated in their bioactivity, the exact structure–activity relationships require further elucidation. For instance, the contribution of specific phytochemicals (e.g., saponins or flavonoids) from *Ziziphi Spinosae Semen* to the final properties of Z-CDs warrants investigation. Second, the pharmacokinetics and biodistribution of Z-CDs in vivo need detailed characterization to optimize dosing regimens. Third, the long-term effects of Z-CDs and their potential interactions with other sepsis therapies (e.g., antibiotics) should be explored. Finally, while the NF-κB pathway was identified as a key target, additional pathways such as Nrf2/ARE, which regulates antioxidant responses, may also contribute to the therapeutic effects and merit further study.

## Conclusions

In this study, we successfully synthesized and characterized the Z-CDs from natural botanical constituents. Z-CDs exhibited significant antioxidant properties and free radical scavenging capabilities for the presence of unsaturated functional groups. Notably, Z-CDs showed a noteworthy therapeutic benefit in mouse and zebrafish models in vivo, with excellent biocompatibility and safety, suggesting substantial potential for medical translation. In mechanism, Z-CDs protected kidney tissues by eliminating excess ROS and inhibiting NF-κB-dependent inflammatory pathway. Taken together, this study enhances our understanding of the role of CDs in the treatment of SA-AKI, thereby offering a kind of potential CDs with translational value for SA-AKI therapy.

## Supplementary Information

Supplementary Material contains supporting figures, additional methodological details, and extended datasets (Fig. S1-S6, Table S1-S2) are provided in this study

## Data Availability

The data that support the findings of this study are available from the corresponding author upon reasonable request.
