# Supplementary material for: Therapeutic potential of naturally derived carbon dots in sepsis-associated acute kidney injury
Source: Chin Med. 2025 Apr 11;20:49. doi: 10.1186/s13020-025-01103-3 (PMC11992765; doi:10.1186/s13020-025-01103-3)
Supplement: Supplementary file 1 — Supplementary Material contains supporting figures, additional methodological details, and extended datasets (Fig. S1-S6, Table S1-S2) are provided in this study [file 13020_2025_1103_MOESM1_ESM.docx]

**Supplemental Information**

**Therapeutic Potential of** **Naturally Derived Carbon Dots in Sepsis-Associated Acute Kidney Injury**

Lei Wang^a#^, Zhong-Yao Li^a#^, Chong-Lei Zhong^a#^, Zi-Yang Teng^a^, Bin Wang^b^, Asma Rehman^c^, Li-Wen Han^a^, Ke-Wu Zeng^d^*, Ji-Guo Zhang^a^*, Zhi-Yuan Lu^a^*

^a^School of Pharmaceutical Sciences & Institute of Materia Medica, State Key Laboratory of Advanced Drug Delivery and Release Systems, Medical Science and Technology Innovation Center, Shandong First Medical University & Shandong Academy of Medical Sciences, Jinan, 250117, China.

^b^Department of Andrology, Guang'anmen Hospital, Chinese Academy of Chinese Medical Sciences, Beijing 100053, China.

^c^National Institute for Biotechnology & Genetic Engineering College Pakistan Institute of Engineering & Applied Sciences (NIBGE-C, PIEAS), Faisalabad 38000, Pakistan.

^d^State Key Laboratory of Natural and Biomimetic Drugs, School of Pharmaceutical Sciences, Peking University, Beijing 100191, China.

#These authors contributed equally to this work.

*Corresponding authors.

[ZKW@bjmu.edu.cn](mailto:ZKW@bjmu.edu.cn) (Ke-Wu Zeng); [jgzhang@sdfmu.edu.cn](mailto:jgzhang@sdfmu.edu.cn) (Ji-Guo Zhang); [luzhiyuan@sdfmu.edu.cn](mailto:luzhiyuan@sdfmu.edu.cn) (Zhi-Yuan Lu)

**Contents**

**1. Supplementary Methods**

**2.** **Supplementary Figures**

**Figure S1. Flowchart of synthesis of Z-CDs.**

**Figure S2. The 3D fluorescence spectra of Z-CDs.**

**Figure S3. Stability of Z-CDs at different NaCl concentrations.**

**Figure S4. Time-dependent fluorescence images of RAW264.7 cells incubated with Z-CDs (400 μg/mL).**

**Figure S5. Experimental design for Z-CDs strategy in CuSO4 on zebrafish model.**

**Figure S6. Z-CDs attenuate H_2_O_2_-induced oxidative damage in HK-2 cells**

**3. Supplementary Tables**

**Table S1. Antibodies and commercial reagents used in this study.**

**Table S2. Primer sequences used in this study.**

1. **Supplementary Methods**

**Cell Culture**

RAW264.7, HUVEC, and HK-2 cells were obtained from Procell Life Science (Wuhan, Hubei, China), and were maintained in a recommended medium by ATCC which was supplied with 10% fetal bovine serum (APExBIO, Houston, TX, USA) and 1% penicillin-streptomycin (Abbkine, Wuhan, Hubei, China)

**Cellular uptake assay**

RAW264.7 cells were seeded in confocal dishes at a density of 5.0 × 10^4^ per well. Subsequently, the cells were incubated in 1 mL of phenol-free DMEM with 10% FBS and 400 μg/mL of Z-CDs for 0, 5, 10, 15, 30, and 60 min. After washing twice with PBS, the images were captured using an LSM980 confocal microscope (Carl Zeiss AG, Oberkochen, Germany) at an excitation wavelength of 350 nm.

**CCK-8 assay**

After being cultured overnight at a density of 1 × 10^5^ in 48-well plates, cells were treated with Z-CDs for 24 h. Cell viability was evaluated using the Cell Counting assay Kit-8 (CCK8) (TargetMol, Shanghai, China).

**Nitric oxide (NO) assay**

The production of NO was assessed from the cell supernatant using an NO assay kit from Nanjing Jiancheng Bioengineering Institute (Nanjing, Jiangsu, China). RAW264.7 cells were exposed to LPS (1 μg/mL) with or without Z-CDs (100, 200, and 400 μg/mL) for 24 h. Cell culture supernatants were gathered and measured with assay kits, following the manufacturer's directions.

**Quantitative PCR**

The procedures for RNA extraction, cDNA synthesis, and qRT-PCR were followed as previously described.(1) To summarize, total RNA was isolated using TRIzol™ Reagent (Thermo Scientific, Waltham, MA, USA) and cDNA was synthesized using the HiScript III RT SuperMix reagent Kit (Vazyme, Nanjing, Jiangsu, China). The qRT-PCR assay was then carried out on a QuantStudio 3 from Thermo Scientific (Waltham, MA, USA). The comparative CT (ΔΔCT) method was used to evaluate the relative expression of the specified genes, normalized to 18S rRNA. The primers used in this study are provided in Supplemental Table S2.

**Western blotting**

The examination of proteins in RAW264.7 cells was performed as described earlier.(2) In brief, cell lysis was performed using the RIPA buffer containing protease and phosphatase inhibitors (Roche, Basel, Switzerland). Proteins were separated via SDS-PAGE and then transferred to a PVDF membrane (Merck Millipore, Darmstadt, Germany). The membrane underwent blocking with 5% BSA for one hour at room temperature and was then incubated with primary antibodies for 12 h at 4 °C. After a one-hour incubation with HRP-conjugated secondary antibodies, the blots were visualized using an ECL substrate. The antibodies utilized for immunoblots are detailed in Supplemental Table S1.

1. **Supplementary Figures**

**Figure S1. Flowchart of synthesis of Z-CDs.**


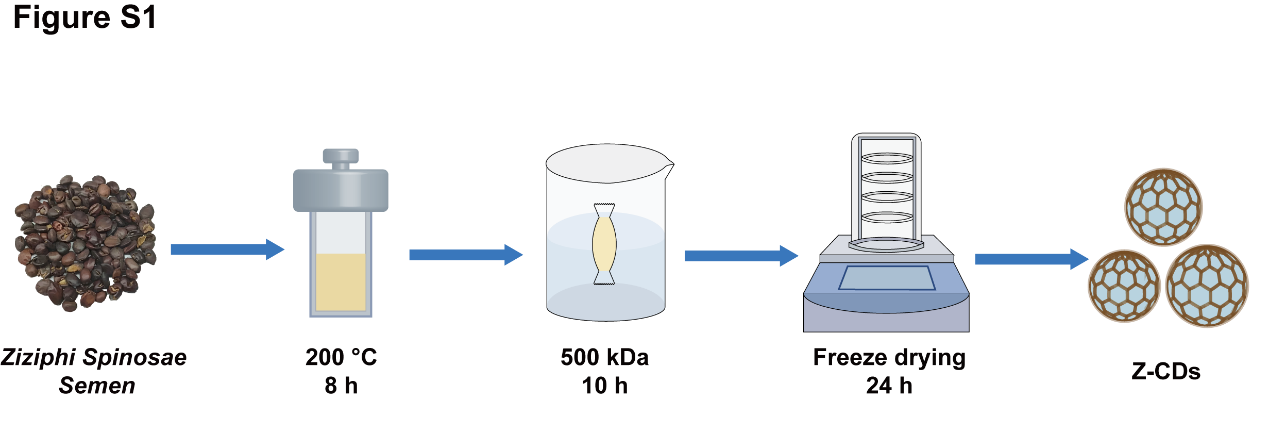


**Figure S2. The 3D fluorescence spectra of Z-CDs.**


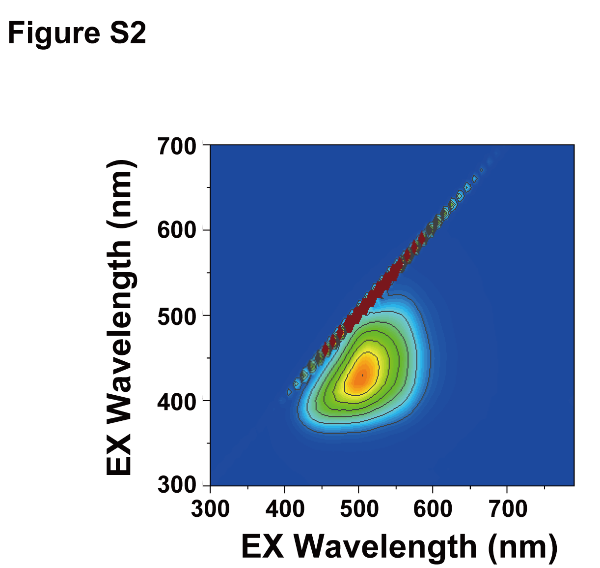


**Figure S3. Stability of Z-CDs at different NaCl concentrations.**


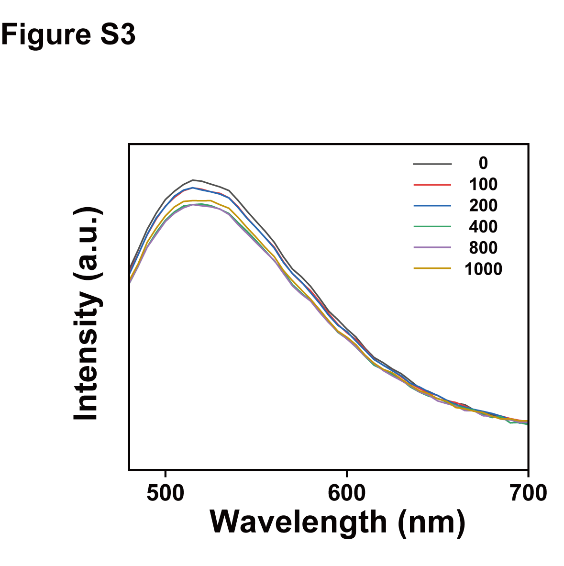


**Figure S4. Time-dependent fluorescence images of RAW264.7 cells incubated with Z-CDs (400 μg/mL).**


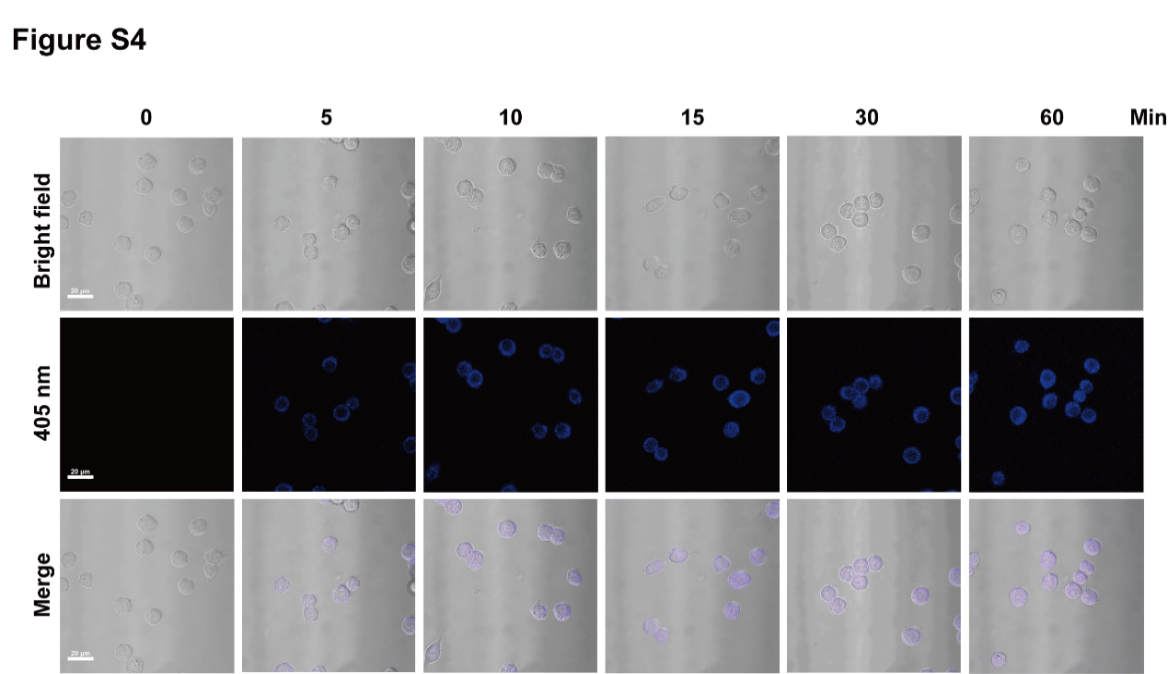


**Figure S5. Experimental design for Z-CDs strategy in CuSO_4_ on zebrafish model.**


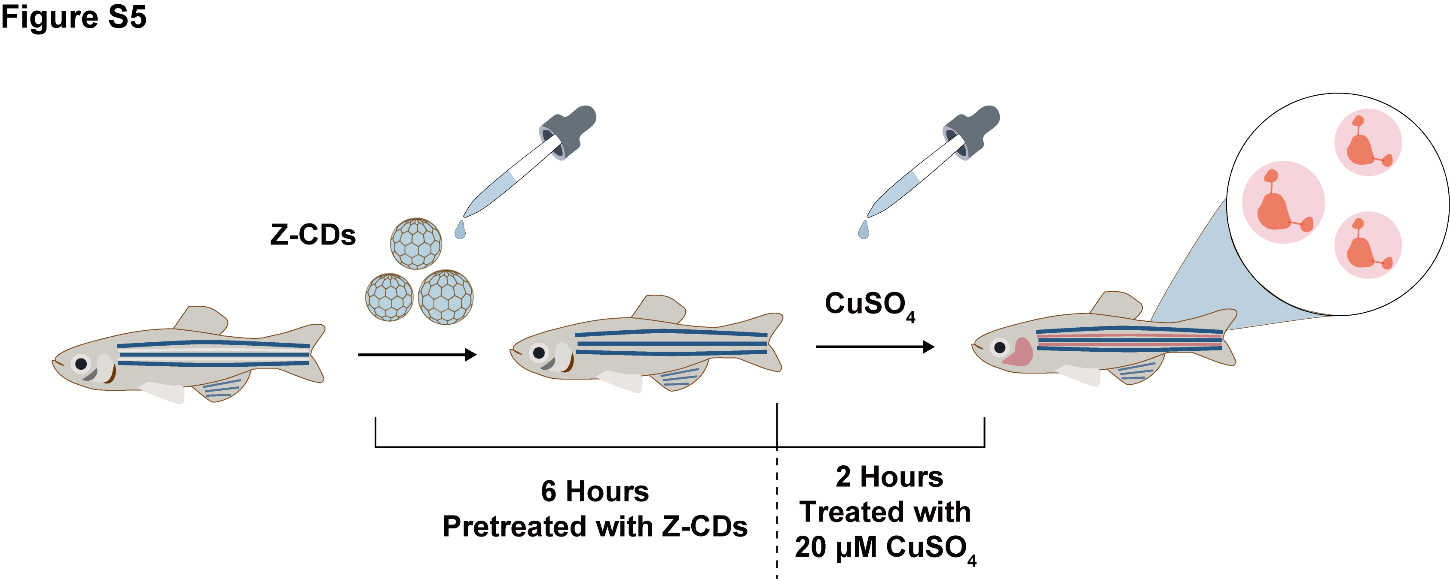


**Figure S6.** **Z-CDs attenuate H_2_O_2_-induced oxidative damage in HK-2 cells.**


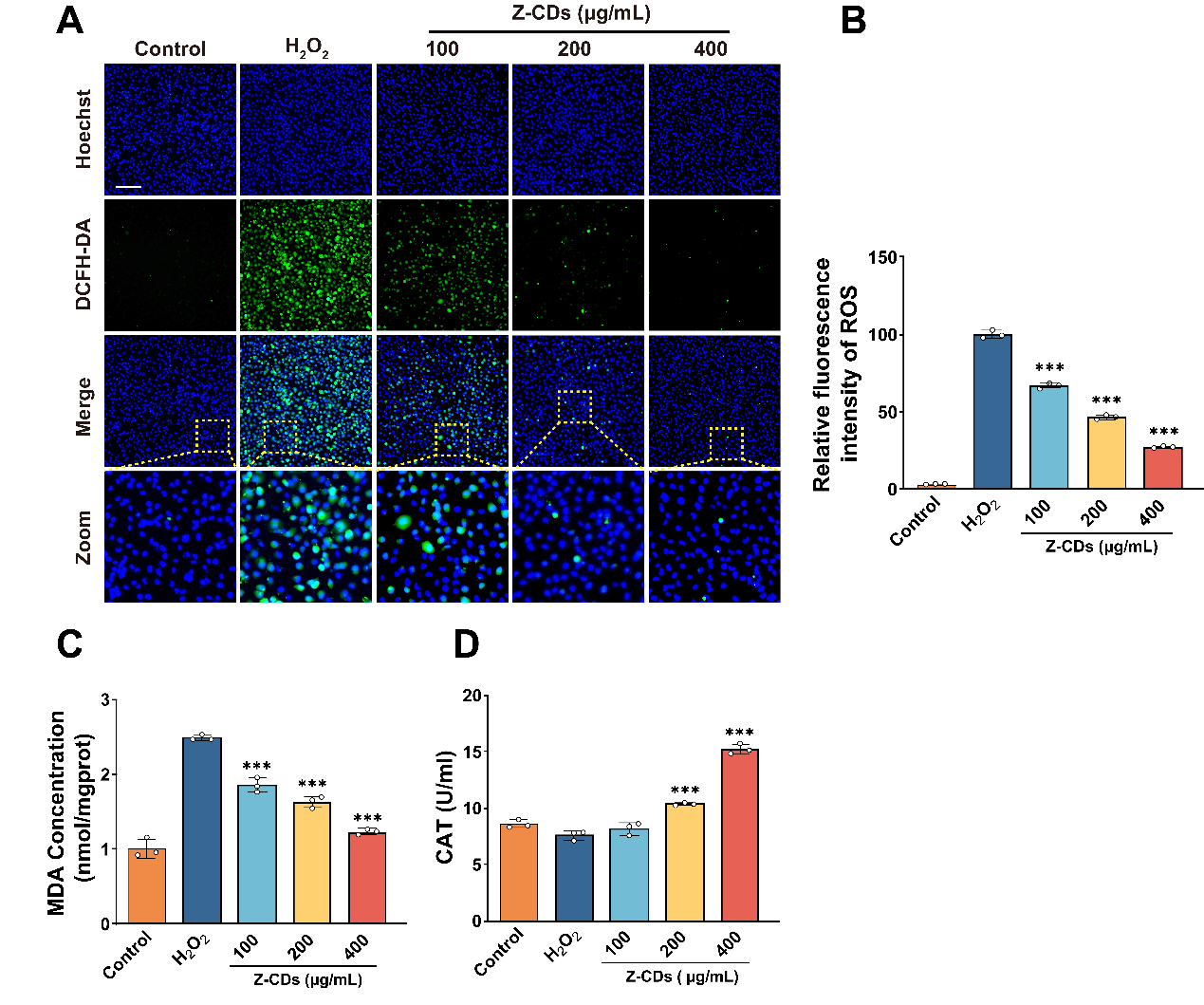


(A-B) Detection of intracellular ROS production by DCHF-DA in HK-2 cells after the treatment of indicated Z-CDs for 24 h. Scale bar: 20 μm. (C) Quantitative analysis of MDA levels. (D) Quantitative analysis of CAT activities. Data are pressed as mean ± SD. **P* < 0.05, ***P* < 0.01, ****P* < 0.001.

1. **Supplemental Tables**

**Table S1. Antibodies and commercial reagents used in this study.**

| **Antibodies/Regents** | **Source** | **Catalog Number** |
| --- | --- | --- |
| GAPDH | Proteintech | 1E6D9 |
| iNOS | Abcam | ab178945 |
| NLRP3 | Proteintech | 68102-1-Ig |
| COX2 | Proteintech | 12375-1-AP |
| p65 | Proteintech | 10745-1-AP |
| p-p65 | CST | #3033 |
| IκBα | Proteintech | 10268-1-AP |
| p-IκBα | CST | #2859 |
| HRP-conjugated Goat Anti-Rabbit IgG(H+L) | Proteintech | SA00001-2 |
| T-AOC Assay Kit | Beyotime | S0119 |
| DCFH-DA | Ambeed | A439906 |
| Mitosox Red | ThermoFisher scientific | M36008 |
| MDA assay kit | Nanjing Jiancheng Bioengineering Institute | A003-4-1 |
| NO assay kit | Nanjing Jiancheng Bioengineering Institute | A013-2-1 |
| HiScript III RT SuperMix | Vazyme | R323-01 |
| ChamQ Universal SYBR qPCR Master Mix | Vazyme | Q711-02 |
| LPS | Sigma | L2880 |
| NBT | Rhawn | R031684 |
| Riboflavin | Rhawn | R055090 |
| L-Methionine | Rhawn | R130487 |
| CuSO_4_ | Rhawn | R019744 |
| TMB | Rhawn | R002584 |
| Ibuprofen | Rhawn | R022654 |
| KBr | Rhawn | R015538 |
| FeSO_4_•7H_2_O | Rhawn | R010224 |
| NaAc-HAc | Rhawn | R139083 |

**Table S2. Primer sequences used in this study.**

| **Genes** | **Oligonucleotides** |
| --- | --- |
| IL-6 | Forward：5’-TGATGCACTTGCAGAAAACA-3’  Reverse：5’-ACCAGAGGAAATTTTCAATAGGC-3’ |
| IL-1β | Forward：5’-GCAACTGTTCCTGAACTCAACT-3’  Reverse：5’-ATCTTTTGGGGTCCGTCAACT-3’ |
| TNF-α | Forward：5’-CCCTCACACTCAGATCATCTTCT-3’  Reverse：5’-GCTACGACGTGGGCTACAG-3’ |
| 18s RNA primer | Forward：5’-GCAATTATTCCCCATGAACG-3’  Reverse：5’-GGCCTCACTAAACCATCCAA-3’ |

**References**

**1**. Z. Lu; H. Yu; Y. Li; G. Xu; X. Li; Y. Liu; Y. Shen; Z. Cai; B. Zhao. Phosphatase, Mg(2+)/Mn(2+) dependent 1B regulates the hematopoietic stem cells homeostasis via the Wnt/β-catenin signaling. *Haematologica.* **2024**, *109* (7), 2144-2156, DOI: 10.3324/haematol.2023.284305

**2**. Z. Lu; L. Huang; Y. Li; Y. Xu; R. Zhang; Q. Zhou; Q. Sun; Y. Lu; J. Chen; Y. Shen; J. Li; B. Zhao. Fine-Tuning of Cholesterol Homeostasis Controls Erythroid Differentiation. *Adv Sci (Weinh).* **2022**, *9* (2), e2102669, DOI: 10.1002/advs.202102669
